# Epidemiological analysis of a COVID-19 outbreak associated with an infected surgeon

**DOI:** 10.1017/S0950268821000650

**Published:** 2021-03-25

**Authors:** Oreste Gallo, Adriano Peris, Michele Trotta, Pietro Orlando, Giandomenico Maggiore, Maria Cilona, Massimo Trovati, Luca Giovanni Locatello

**Affiliations:** 1 Department of Otorhinolaryngology, Careggi University Hospital, Largo Brambilla, 3 - 50134 Florence, Italy; 2 Department of Experimental and Clinical Medicine, University of Florence, Italy; 3 Department of Emergency, Intensive Care Unit and Regional ECMO Referral Centre, Careggi University Hospital, Largo Brambilla, 3 - 50134 Florence, Italy; 4 Department of Infectious and Tropical Diseases, Careggi University Hospital, Largo Brambilla, 3 - 50134 Florence, Italy

**Keywords:** COVID-19, head and neck surgery, hospital infection, otorhinolaryngology, surgical procedures

## Abstract

Control of the novel COronaVIrus Disease-2019 (COVID-19) in a hospital setting is a priority. A COVID-19-infected surgeon performed surgical activities before being tested. An exposure risk classification was applied to the identified exposed subjects and high- and medium-risk contacts underwent active symptom monitoring for 14 days at home. All healthcare professionals (HCPs) were tested for severe acute respiratory syndrome-coronavirus-2 (SARS-CoV-2) at the end of the quarantine and serological tests were performed. Three household contacts and 20 HCPs were identified as high- or medium-risk contacts and underwent a 14-day quarantine. Fourteen HCPs and 19 patients were instead classified as low risk. All the contacts remained asymptomatic and all HCPs tested negative for SARS-CoV-2. About 25–28 days after their last exposure, HCPs underwent serological testing and two of them had positive IgM but negative confirmatory swabs. In a low COVID-19 burden area, the in-hospital transmission of SARS-CoV-2 from an infectious doctor did not occur and, despite multiple and frequent contacts, a hospital outbreak was avoided. This may be linked to the adoption of specific recommendations and to the use of standard personal protective equipment by HCPs.

The CoronaVIrus Disease 2019 (COVID-19) caused by severe acute respiratory syndrome-coronavirus-2 (SARS-CoV-2) is still affecting the whole planet, with over 110 million confirmed cases and 2.4 million related deaths (WHO Dashboard, 20th February 2021). Several studies have shed light on the infection dynamics at the population level, but still little is known regarding the in-hospital transmission, although hospitals and healthcare facilities have been acting as epicentres of the pandemic [1]. There is an urgent need to better characterise hospital-associated SARS-CoV-2 diffusion and it seems clear that strict control measures are necessary to prevent nosocomial transmission from patients to healthcare professionals (HCPs), as well as from HCPs to others.

Herein, we report the dynamics of how an infected otorhinolaryngologist, who performed inpatient and surgical activity during the window period and during his first symptomatic days in an Italian University hospital located in Florence, Tuscany, did not provoke a hospital outbreak. For the infected surgeon, the initial exposure to the virus is assumed to have occurred around 26 February 2020, in the very early weeks of the so-called ‘first wave’. At that time, Tuscany was not considered a ‘red zone’, as only a limited number of SARS-CoV-2 infections were detected in the area. However, after Italian Public Health authorities' advice, HCPs were asked to wash their hands with an alcoholic solution after each visit and to use basic personal protective equipment (PPE, simple surgical facemasks and disposable gloves), as most of the head and neck procedures are known to possibly aerosolise droplets; moreover, there should be as few people as possible in the same room at any one time. However, whereas all the staff always used basic PPE when dealing with patients, we admit they did not during meetings or in shared environments where only distancing measures were taken. Another critical issue was constituted by the lack of any triage protocol, especially for external consultations because neither formal screening programme was conducted nor thermoscans or other filtering measures were available. Furthermore, systematic testing for SARS-CoV-2 was not immediately implemented, in particular in terms of surgical pathways.

For research aims, the date of symptoms onset for the index surgeon is considered as the day of investigation 1 (DOI 1), while ‘contacts’ were defined as people who reported or were identified to have had potential exposure to the index on or after DOI 1.

The index case is a 61-year-old male who spent 9 days in Northern Italy until 1 March 2020. He has a personal history of arterial hypertension, previous myocardial infarction and asthma. He drinks alcohol occasionally and he has never smoked. His medications include clopidogrel, atorvastatin and ramipril/hydrochlorothiazide. He came back to work on 2 March 2020 (DOI 1) and he took part in major oncological surgical procedures. A fluctuating, mild, dry cough was the only symptom he complained of on those four days, but this was attributed to his long history of asthma. On the night between 5 and 6 March (DOI 4-5), the index physician started to feel ill and decided to rest at home for the next few days. His clinical conditions quickly worsened and he went to the Emergency Department on 8 March (DOI 7). Chest radiography showed bilateral reticular and patchy infiltrates. Sputum, oropharyngeal and nasopharyngeal swabs were obtained and all the specimens tested positive for SARS-CoV-2. He was promptly hospitalised and, in the following days, he developed respiratory distress that required endotracheal intubation and mechanical ventilation, so he was transferred to the intensive care unit (ICU) on DOI 10. On DOI 19, a worsening of gas exchange was noticed. A computed tomography pulmonary angiography revealed acute pulmonary embolism and the dosage of enoxaparin was therefore doubled. Subsequently, clinical status gradually improved and FiO_2_ was gradually reduced. As from DOI 27, the surgeon was no longer sedated and only required low flow oxygen. Tracheostomy was removed on DOI 32 and he was finally discharged from the ICU after two consecutive (DOI 32 and DOI 33) negative nasopharyngeal swabs.

Immediately after index case confirmation, a prompt epidemiological investigation was undertaken to track all the in-hospital and familiar contacts. All the movements, encounters and clinical and surgical activities performed by the physician from DOI 1 to the day of COVID-19 diagnosis (DOI 6) were retrospectively investigated, in order to pinpoint as many contacts as possible. Hospital records were reviewed to identify all the patients that attended our Department for in-hospital examination and surgical interventions. All HCPs who worked on the same floor of the building were tracked and interviewed to evaluate individual exposure, as well as the three persons who lived with him. From DOI 6, epidemiological data were prospectively collected by the first author.

Contacts' exposure risk was stratified according to the three-tiered classification system which was proposed by the Region of Tuscany COVID-19 Emergency Task Force, and that was in force at the time of the virological diagnosis [2]. Such criteria have been translated into the English language by the authors and are reported in Table 1. For research purposes, we also decided to retrospectively reclassify all the contacts enrolled in the present study on the basis of the latest Interim Guidance of the United States Centers for Disease Control and Prevention (CDC) for risk assessment after potential exposure to COVID-19 [3, 4]. Although primarily developed for HCPs only, these criteria were conversely applied to non-COVID-19 patients who were exposed to index physician and that were assumed not to have had prolonged contact. On the other hand, to evaluate HCPs exposure risk, CDC frameworks designed for community members were considered preferable. In fact, the index doctor shared the same rooms with other HCPs for at least 7 h per day during their work shifts; such criteria can account for a much longer period of time as if they were living with each other [3, 4].

**Table 1.** Exposure risk criteria used in this study, contacts involved and a comparison with the CDC recommendations [2–4]

Exposure risk group	Region of Tuscany criteria for HCPs exposure	HC	HCP	Pts	Tot	CDC criteria for community exposure	CDC criteria for HCPs exposure	HC	HCPs	Pts	Tot
**High risk**	Anybody who directly assists a COVID-19 case or handle patient's biological samples with a direct cutaneous or mucous exposition to patient's biological material; puncture or penetrating wound with potentially contaminated material; corpse handling or recomposition without adequate PPE.	0	0	0	0	Living in the same household as, being an intimate partner of, or providing care in a non-healthcare setting (such as a home) for a person with symptomatic and laboratory-confirmed COVID-19 infection without using recommended precautions for home care and home isolation.	HCPs wearing no PPE or not wearing facemask or respirator and having a prolonged close contact with a COVID-19 patient who was not wearing a facemask.	3	13	0	16
**Medium risk**	Anybody who directly assist a COVID-19 case or handle patient's biological samples without recommended or with accidentally damaged PPE, without direct exposition to biological patient's material; anybody who, without PPE, had face-to-face contact with a COVID-19 case devoid of a mask, longer than 15 min at a distance lower than 2 m; anybody who, without PPE, stayed in an enclosed space with a COVID-19 case devoid of a mask, longer than 15 min at a distance lower than 2 m.	3	20	0	23	Close contact with a person with symptomatic laboratory-confirmed COVID-19; on an aircraft, being seated within 6 feet (2 m) of a traveller with symptomatic laboratory-confirmed COVID-19 infection; living in the same household as, an intimate partner of, or caring for a person in a non-healthcare setting (such as a home) to a person with symptomatic laboratory-confirmed COVID-19 infection while consistently using recommended precautions for home care and home isolation.	HCPs wearing no PPE or not wearing facemask or respirator having prolonged close contact with a COVID-19 patient who was wearing a facemask; HCPs not wearing eye protection having prolonged close contact with a COVID-19 patient who was not wearing a facemask.	0	7	2	9
**Low risk**	Anybody who had a contact with a COVID-19 case without using PPE, with a patient wearing a mask; anybody who, wearing recommended PPE, directly assist a COVID-19 case or handle the patient's biological samples.	0	14	19	33	Being in the same indoor environment (e.g. a classroom, a hospital waiting room) as a person with symptomatic laboratory-confirmed COVID-19 for a prolonged period of time but not meeting the definition of close contact.	HCPs wearing all recommended PPE (except wearing a facemask instead of a respirator) or not wearing eye protection or gloves or gown having a prolonged close contact with a COVID-19 patient with a facemask; HCP wearing all recommended PPE (except wearing a facemask instead of a respirator) or not wearing gloves or gown having prolonged close contact with a COVID-19 patient without a facemask.	0	14	17	31
**Total**		3	34	19	56			3	34	19	56

Abbreviations: COVID-19, COronaVIrus Disease 2019; CDC, Centers for Disease Control and prevention; HC, household contacts; HCPs, health-care professionals; PPE, personal protective equipment; Pts, patients; Tot, total.

The analysis identified 56 contacts that were staged for exposure risk as explained above (Table 1). Considering the cumulative exposure risk, 5 contacts (8.9%) were exposed for 4 days, 7 (12.5%) for 3 days, 10 (17.9%) for 2 days and 34 (60.7%) for 1 day (Supplementary Figure). A detailed description of the surgeon's activities and of the non-HCPs contacts is given in the Supplementary Appendix. Twenty-three of the 56 contacts (41%) were included in the ‘medium risk’ group that was composed of 6 physicians, 4 residents, 9 nurses and 3 physician's relatives. As established by the Italian Ministry of Health, for all the members in the high- or medium-risk group, an active monitoring protocol and a quarantine period were adopted. They were obliged to stay at home, measuring body temperature at least twice a day, and to immediately alert the Public Health Authorities in the case of appearance of any possible COVID-19 symptoms (fever, cough, tiredness, dyspnoea, rhinorrhoea, diarrhoea, dysgeusia and anosmia/hyposmia). Pharyngeal swabs could be obtained in case of symptoms appearance or, conversely, on the 15th day after the last remembered contact with the index (DOI 1−4). Strikingly, nobody ever complained of any COVID-19-associated symptoms and they all obtained pharyngeal swab on the 15th day. 23 of 23 tested negative on reverse transcription-polymerase chain reaction assay and could come back to work. Regarding the three index doctor's household contacts (one of whom had stayed in a high-risk zone with him) none developed any symptoms, and swabs were thus not retrieved.

Thirty-three persons (69%) were instead classified as ‘low-risk’ contacts. In this group, there were 10 physicians belonging to other departments, 4 nurses and 19 patients. All the patients who had close contact with the index doctor in the period 2−5 March were classified as ‘low-risk’ because he performed all procedures (e.g. oral cavity inspection and laryngoscopy) while wearing appropriate PPE (surgical mask and disposable gloves). Furthermore, apart from physical examination, everyone kept a distance of at least 2 m from one another. HCPs in the low-risk group were allowed by the hospital's authorities to keep on working with PPE and strict protection measures. Moreover, they were obliged to measure body temperature twice a day for 14 days and to alert if any of the aforementioned symptoms developed. None of them complained of any symptoms during the following 2 weeks. Finally, low-risk non-HCPs contacts were invited to stay at home taking the usual precautions and to alert in case of any symptom onset during the subsequent 14 days. Nobody called and, on 26 March, 16 of 19 non-HCPs contacts were all phoned back again to assess their clinical condition in the previous two weeks by a standardised questionnaire. Even in this case, none complained of any COVID-19-associated manifestations. Since the three remaining patients were already scheduled for imminent surgery, it was decided to test one patient on 23 March (DOI 22) and two patients on 1 April (DOI 31). All the samples eventually tested negative and patients could undergo surgery.

Regardless of the initially assigned exposure risk category, on DOI 26, 29 HCPs obtained serological analysis. NADAL^®^ COVID-19 IgG/IgM test (nal von minden GmbH, Moers, Germany), a qualitative chromatographic lateral flow immunoassay was used for anti-SARSCoV-2 IgG and IgM detection. Among the HCPs included in the medium-risk group, one individual (0.5% if we consider the Tuscany region classification, 14.3% according to CDC criteria) had positive IgM results (++−−) with no detectable IgG. In the low-risk group, only a weak positivity in IgM (+−−−) was detected in one nurse (7.1% according to both classifications). Both the contacts obtained two confirmatory nasopharyngeal and oropharyngeal swabs, 24 h-distanced from one another, on DOI 31 and DOI 32, but no viral RNA was detected. Nevertheless, all the people with a positive or uncertain serological result were further obliged to 14 days of quarantine with serial control swabs.

In this paper, we have presented a detailed examination of a potential COVID-19 hospital outbreak caused by an infected working surgeon. Actually, neither the patients nor the HCPs who worked with the COVID-19-positive surgeon were apparently infected. During his whole working period, the surgeon was always wearing a simple facemask and it is conceivable that such simple PPE may have been protective both for the patients and the other HCPs [1]. Our analysis ultimately suggests that doctor-to-patient diffusion, even during surgical procedures, may be less frequent than patient-to-HCP or person-to-person transmission, as reported in the first reports [5]. Other groups have shown similar experiences in the management of HCPs potentially exposed: Vimercati *et al*., for instance, reported a very low prevalence (0.4%) of positive healthcare workers and this was possible thanks to the application of a general prevention and reporting system [6]. Another very important epidemiological issue remains the choice of the risk classification system: besides the aforementioned CDC criteria, a simple dichotomous classification according to the correct use of PPE during the contact with the index case has demonstrated to be able to reliably identify subjects most at-risk [7]. A very interesting study from South Korea has recently offered a mathematical model of in-hospital transmission dynamics of COVID-19, and the authors showed how an ‘early testing strategy’ for newly hospitalised patients is effective to reduce nosocomial outbreaks. Notably, while in a low transmission context, the detection of positive patients before admission seems the most important preventive measure, the widespread use of PPE is crucial to control viral transmissibility in high transmission settings [8]. The effectiveness of universal masking in the in-hospital setting has been also recently strengthened by studies showing that SARS-CoV-2 positivity rates among HCPs were significantly reduced after its implementation; furthermore, even in large hospitals with nearly 10,000 admitted patients, no clear-cut case of in-hospital acquired SARS-CoV-2 infection was registered [1, 8]. In addition to the triage of suspected cases and PPE use, it was shown that a massive testing strategy, based on nasopharyngeal swabs, to be a third pillar in the control of the pandemic: a group from a highly endemic zone has published its institutional protocol that included molecular testing of all HCPs every 7–10 days, irrespective of their symptoms, and none of the healthcare workers became positive despite being directly in contact with COVID-19 patients [9].

The favourable outcome in this study might also depend on the relatively low COVID-19 cases burden present in our hospital at the time of these events. On 2 March (DOI 1), the number of confirmed COVID-19 cases in the whole Tuscany region was 123, of whom only 23 were admitted to our hospital and we believe our risk estimate is not applicable to settings where a greater proportion of HCPs is infectious. According to the literature, the RNA detection rate in samples collected before day 7 or during days 15−39 after illness onset decreases from 66.7% to 45.5%, while the presence of antibodies increases rapidly to 100% tests would strongly suggest the lack of COVID-19 transmission in exposed HCPs but, because of the many commercially available serological tests, it is not possible to draw any conclusions until more definite evidence is published [10]. Our preliminary study has some limitations. Despite all efforts, it is always hard to perfectly reconstruct the full chain of transmission and it is possible that not all potentially exposed individuals have been identified. In detail, on DOI 1 and DOI 2, the index travelled from his home to the workplace (about a 30-min trip) by public tram, and retrieving these passengers was not feasible.

In conclusion, we have conducted a thorough analysis of a potential surgeon-to-patient and surgeon-to-HCPs transmission of SARS-CoV-2. The absence of COVID-19 RNA and of an antibody response among patients treated by the surgeon as well as among HCPs suggest that distancing measures, hygiene practice and PPE may be very useful for in-hospital control. Future studies, especially in different hospital settings, are needed but at present widespread use of PPE, the rapid isolation and testing of all HCPs potentially infected seems to be the only way to minimise the risk of a hospital outbreak.

## Data Availability

Data are available upon reasonable request to the corresponding author. Gallo: Conceptualisation; Data curation; Formal analysis; Supervision; Methodology; Validation; Visualisation; Writing − review and editing. Peris: Supervision; Validation; Visualisation. Trotta: Conceptualisation; Data curation; Formal analysis; Validation. Orlando: Conceptualisation; Data curation; Formal analysis; Methodology; Supervision; Validation; Visualisation; Roles/Writing − original draft; Writing − review and editing. Maggiore: Supervision; Validation; Visualisation. Cilona: Conceptualisation; Methodology; Data curation; Formal analysis. Trovati: Supervision; Validation; Visualisation; Roles/Writing − original draft; Writing − review and editing. Locatello: Conceptualisation; Data curation; Formal analysis; Methodology; Project administration; Supervision; Validation; Visualisation; Roles/Writing − original draft; Writing − review and editing.

## Supplementary material

For supplementary material accompanying this paper visit http://dx.doi.org/10.1017/S0950268821000650. click here to view supplementary material
