# Supplementary material for: Epidemiological analysis of a COVID-19 outbreak associated with an infected surgeon
Source: Epidemiol Infect. 2021 Mar 25;149:e77. doi: 10.1017/S0950268821000650 (PMC8042382; doi:10.1017/S0950268821000650)
Supplement: Supplementary file 1 [file hygsup.zip › S0950268821000650sup001.docx]

#### **SUPPLEMENTARY APPENDIX 1: A detailed description of non-HCP contacts.**

In order to identify every single non-HCP who possibly had contact with the index case, we established a day-by-day log of the doctor’s activities:

1. On March 2, 2020, he was a member of the surgical team performing a hemiglossectomy with selective neck dissection, for a cT2N0 squamous cell carcinoma of the tongue. The total duration of the surgery was about 4 hours;
2. On March 3, together with the Department’s chief, he visited 11 patients scheduled for the surgery:

- a 70-year-old woman with atherosclerosis scheduled for a parotidectomy because of a pleomorphic adenoma and who did not require transnasal flexible laryngoscopy (TFL-);
- a 75-year-old woman with a history of heavy smoking and scheduled for excision of a Reinke’s edema, and who did receive transnasal flexible laryngoscopy (TFL+);
- a 39-year-old man who underwent TFL before surgery for obstructive sleep apnea syndrome;
- 76-year-old female smokers on anticoagulant therapy and who is scheduled for laryngeal leukoplakia excision (TFL+);
- a 58-year-old woman needing transnasal fistula repair for idiopathic cerebrospinal fluid rhinorrhea (TFL+);
- a 33-year-old man for whom revision surgery was planned after endoscopic resection of inverted sinonasal papilloma (TFL+);
- a 58-year-old man scheduled for functional endoscopic sinus surgery for recurrent bilateral nasal polyposis (TFL+);
- a 52-year-old woman scheduled for an excision of a Warthin’s tumor of the right parotid gland (TFL-);
- a 58-year-old woman with asthma and currently smoking and for which left submandibular gland excision was planned due to a 2 x 1.3 cm mass with suspicious cytology (TFL-);
- a 78-year-old male former smoker, with a history of gastric cancer, atrial fibrillation, and laryngeal cancer and who needs biopsy under direct laryngoscopy because of a suspicious laryngeal lesion (TFL+);
- a 53-year-old female smoker with a history of hepatitis C infection and who had previous radiation therapy plus a tracheostomy for a laryngeal squamous cell carcinoma. She was scheduled for diagnostic panendoscopy under general anesthesia (TFL+);
- a 79-year-old woman with previous acute myocardial infarction, hemiplegic due to cerebrovascular accident and on anticoagulant therapy, and presenting for an rcT4 squamous cell carcinoma of the cheek (TFL+).

1. On March 4, he took part in a transmandibular excision for a p16- cT4aN2b oropharyngeal squamous cell carcinoma, with associated bilateral neck dissection and reconstruction with a pectoralis major myocutaneous flap. In particular, the index doctor acted as the first operator in the tracheostomy, as the second operator for the harvesting of the flap and as the third operator for the rest of the surgery. The total duration of the procedure was about 8 hours;
2. On March 5, he was involved in ear, nose and throat consultations and he visited:

- a 74-year-old man, that was operated on the day before and who was in the sub-intensive unit care
- a 65-year-old woman, who was investigated with flexible laryngoscopy, in an ENT consultation at the Emergency Department because of a newly discovered pharyngeal mass;
- a 28-year-old man who received a drainage procedure for an otohematoma;
- a 65-year-old man, former smoker, and who sent to our attention because of a laryngeal leukoplakia;
- a 62-year-old woman, requiring surgical medications;
- a 36-year-old man, recovered in ICU after a work-related accident, and with an ear-penetrating wound that needed ENT evaluation.
