# Supplementary figures and images for: Epidemiological analysis of a COVID-19 outbreak associated with an infected surgeon
Source: Epidemiol Infect. 2021 Mar 25;149:e77. doi: 10.1017/S0950268821000650 (PMC8042382; doi:10.1017/S0950268821000650)

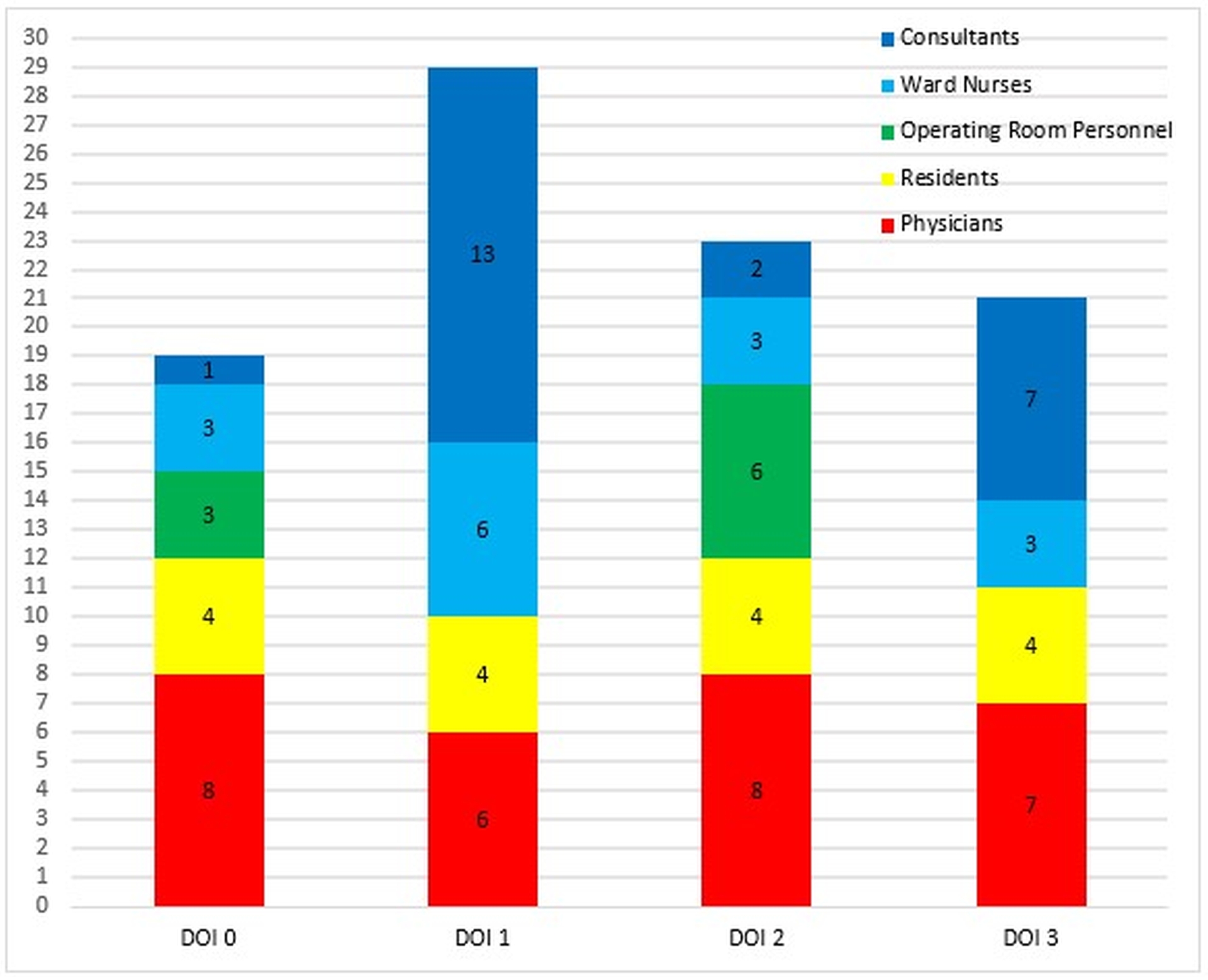

Supplement: Supplementary file 1 [file hygsup.zip › S0950268821000650sup002.jpg]
